# HLA-DRB1 Genotypes and the Risk of Developing Anti Citrullinated Protein Antibody (ACPA) Positive Rheumatoid Arthritis

**DOI:** 10.1371/journal.pone.0064108

**Published:** 2013-05-30

**Authors:** Nathalie Balandraud, Christophe Picard, Denis Reviron, Cyril Landais, Eric Toussirot, Nathalie Lambert, Emmanuel Telle, Caroline Charpin, Daniel Wendling, Etienne Pardoux, Isabelle Auger, Jean Roudier

**Affiliations:** 1 Rhumatologie, Hôpital Sainte Marguerite, Assistance Publique Hôpitaux de Marseille, Marseille, France; 2 Institut National des Sciences et de la Recherche Médicale, Unité Mixte de Recherche en Santé 1097, Aix Marseille Université, Marseille, France; 3 Laboratoire d'Immunogénétique et d'Histocompatibilité, Etablissement Français du Sang Alpes-Méditerranée, Marseille, France; 4 Centre d'Investigation Clinique 506, Hôpital Saint Jacques, Besançon, France; 5 Rhumatologie, Hôpital Minjoz, Besançon, France; 6 Centre National pour la Recherche Scientifique, Unité Mixte de Recherche 6632, Équipe Évolution Biologique et Modélisation, Aix Marseille Université, Marseille, France; South Texas Veterans Health Care System and University Health Science Center San Antonio, United States of America

## Abstract

**Objective:**

To provide a table indicating the risk for developing anti citrullinated protein antibody (ACPA) positive rheumatoid arthritis (RA) according to one’s HLA-DRB1 genotype.

**Methods:**

We HLA-DRB1 genotyped 857 patients with ACPA positive RA and 2178 controls from South Eastern and Eastern France and calculated Odds Ratios (OR) for developing RA for 106 of 132 possible genotypes accounting for 97% of subjects.

**Results:**

HLA-DRB1 genotypic ORs for developing ACPA positive RA range from 28 to 0.19. HLA-DRB1 genotypes with HLA-DRB1*04SE (HLA-DRB1*0404, HLA-DRB1*0405, HLA-DRB1*0408), HLA-DRB1*04∶01, HLA-DRB1*01 are usually associated with high risk for developing RA. The second HLA-DRB1 allele in genotype somewhat modulates shared epitope associated risk. We did not identify any absolutely protective allele. Neither the Reviron, nor the du Montcel models accurately explains our data which are compatible with the shared epitope hypothesis and suggest a dosage effect among shared epitope positive HLA-DRB1 alleles, double dose genotypes carrying higher ORs than single dose genotypes.

**Conclusion:**

HLA-DRB1 genotypic risk for developing ACPA positive RA is influenced by both HLA-DRB1 alleles in genotype. We provide an HLA-DRB1 genotypic risk table for ACPA positive RA.

## Introduction

Susceptibility for developing rheumatoid arthritis (RA) is associated with particular HLA-DRB1 alleles like HLA-DRB1*04, HLA-DRB1*01 and HLA-DRB1*10 [1]. A molecular basis for this association was provided by Gregersen and al. who showed that RA associated HLA-DRB1 alleles contain a conserved 5 amino acid stretch, the “shared epitope” (SE) in the third hypervariable region of their DRB1 chain [2]. This lead to a simple model in which shared epitope positive HLA-DRB1 alleles carried susceptibility for developing RA and shared epitope negative alleles were considered neutral.

Since 1987, numerous studies have confirmed the association of RA with shared epitope positive HLA-DRB1 alleles. However, it has been suggested that a few shared epitope negative HLA-DRB1 alleles protect against the development of RA, whereas most others are neutral [3]–[5].

Different models have been proposed to predict whether a given genotype (ie both genes present in an individual) will be susceptible, neutral or protective towards the development of RA. These models are based on the classification of HLA-DRB1 alleles in different categories depending on the sequence of their third hypervariable region [3]–[5]. The Reviron model proposes that shared epitope positive HLA-DRB1 alleles which have a very positive charge in their P4 pocket (HLA-DRB1*0401, HLA-DRB1*0404, HLA-DRB1*0405, HLA-DRB1*0408, HLA-DRB1*0101) predispose to RA whereas among shared epitope negative alleles, those which have a positive charge in their P4 pocket, called XP4p (HLA-DRB1*03, HLA-DRB1*09, HLA-DRB1*14, HLA-DRB1*15, HLA-DRB1*16), are neutral and those with a negative or neutral charge in their P4 pocket, called XP4n (HLA-DRB1*07, HLA-DRB1 *08, HLA-DRB1*11, HLA-DRB1*0402), protect against RA [4]. Sophie Tézenas du Montcel proposed a model which considers 4 groups of HLA-DRB1 alleles according to the sequence of their third hypervariable region: S2 (QKRAA), S3P (QRRAA, RRRAA), S3D (DRRAA), S1 (ARAA and ERAA) and X (non RAA). In the du Montcel model, S2 alleles confer very high risk, S3P alleles confer high risk and S1, S3D and X alleles confer low risk to develop RA [5]. The main difference between the two models is the absence of protective alleles in the du Montcel model.

However, the validity of these models has not been established. Here, we decided to calculate the risk for developing RA given by any individual HLA-DRB1 genotype, with no a priori model.

Complexity of the RA/HLA-DRB1 association further increased with the discovery that RA can be divided into two subtypes according to the presence in a patient’s serum of autoantibodies directed at citrullin residues on different proteins [6]. Indeed, the sera of two thirds of patients with RA contain antibodies to citrullinated proteins. Citrullin is an amino acid generated by the posttranslational modification of arginyl residues by peptidyl arginine deiminases. These autoantibodies are called anti citrullinated protein antibodies (ACPA) [6]. Presence or absence of ACPAs define two subtypes of RA. ACPA positive RA is well defined and the 2010 ACR criteria for the diagnosis of RA include a positive ACPA test [7]. ACPA negative RA is much more heterogeneous [8]. The association of RA with shared epitope positive HLA-DRB1 alleles is stronger in ACPA positive RA than in ACPA negative RA [8], [9].

Here, we studied a series of 857 ACPA positive RA patients and compared them with a series of 2178 controls.

Every patient and control, all from South Eastern France was HLA-DR typed for 20 different HLA-DRB1 alleles. Bayesian statistics were used to define susceptible and protective genotypes and to calculate accurate confidence intervals for the associated Odds Ratios. We calculated Odds Ratios (OR) for 102 of 136 possible HLA-DRB1 genotypes, for which the number of patients plus controls was at least 5. Thirty genotypes had ORs significantly higher than 1 and were considered high risk, 45 had ORs not significantly different from 1 and were considered neutral, 27 genotypes had ORs significantly lower than 1 and were considered low risk.

## Patients and Methods

### Patients and Controls

Ethical approval was obtained for this study from CPP Marseille II. All participants gave their informed consent. All participants signed informed consent according to the Declaration of Helsinki.

In this cohort study, we took blood samples from 857 ACPA positive RA patients and 2178 controls from Marseilles (South Eastern France) and Besançon (Eastern France). RA patients fulfilled the 2010 EULAR/ACR criteria [7] and tested positive for anti cyclic-citrullinated peptide antibodies (ACPA) using the anti CCP2 kit which is routinely used in our patients with arthritis. The control group included voluntary blood or bone marrow donors from the same area. Sex ratio and age were not significantly different between patients and controls. Seventy one percent of patients were positive for at least one shared epitope positive HLA-DRB1 allele (Table 1).

**Table 1:** Patients' and controls' characteristics.

	Patients	Controls
Number	857	2178
Female	74.40%	69%
Male	26%	31%
ACPA	100%	0%
RF pos	91%	NC
SE double dose	20.40%	4.50%
SE single dose	51%	37%
Age at diagnosis	43.6	42

RF: rheumatoid factor, pos: positive, SE: Shared Epitope, ACPA: anti citrullinated protein antibodies, NT: not tested

### ACPA and Rheumatoid Factor Testing

Positivity for anti citrullinated peptide antibodies (ACPA) was used to define mainstream, classical RA [8]. ACPA were detected by anti CCP2 Enzyme-linked immuno sorbent assay (ELISA) (Immunoscan RA, Euro-Diagnostica, Arnhem, the Netherlands). This kit is currently the most commonly used in Southern France and positivity is defined by a cutoff value of 25 Units/ml at a dilution of 1/50. 91% of our patients selected for ACPA positivity by anti CCP2 testing tested positive for rheumatoid factor by ELISA using the Orgentec Kit (Mainz, Germany) with a 20 Units/ml cutoff at a 1/100 dilution.

### HLA-DRB1 Genotyping

Low resolution HLA-DRB1 typing was carried out according to the manufacturer's specification for LABType SSO (One Lambda Inc, USA) and the retrieved output was analyzed by HLA Fusion v 1.2.1. software (One Lambda Inc, USA) for allele identification. HLA-DRB1*04-positive samples were subtyped by PCR sequence-specific primers (Olerup SSP HLA-DRB1*04, Genovision, Vienna, Austria).

We typed for the following alleles or group of alleles: HLA-DRB1*01, HLA-DRB1*03, HLA-DRB1*04∶01, HLA-DRB1*04∶02, HLA-DRB1*04∶03, HLA-DRB1*04∶04**,** HLA-DRB1*04∶05, HLA-DRB1*04∶06, HLA-DRB1*04∶07, HLA-DRB1*04∶08, HLA-DRB1*07, HLA-DRB1*08, HLA-DRB1*09, HLA-DRB1*1001, HLA-DRB1*11, HLA-DRB1*12, HLA-DRB1*13, HLA-DRB1*14, HLA-DRB1*15, HLA-DRB1*16. HLA-DRB1*04∶04**,** HLA-DRB1*04∶05 and HLA-DRB104∶08 were considered as one allelic group called HLA-DRB1* 04SE. The rationale for this regrouping is that these three HLA-DRB1*04 alleles encode a common aminoacid sequence, QRRAA in their third hypervariable region (HV3). This HV3 sequence is different from that encoded by HLA-DRB1*0401, QKRAA. Therefore, HLA-DRB1*0401 is not included in this HLA-DRB1*04 allelic subgroup that we called HLA-DRB1*04SE.

After this regrouping, we ended up with 16 allelic groups defining 136 genotypes.

### Statistical Analysis

The statistical method used in this study to estimate the Odds Ratio and its confidence interval for the different genotypes is based on the Bayesian theory. This method is more stringent that the one using the maximization of the likelihood function especially when there are less than 5 individuals in one of the two classes of genotypes (patient or control). This method was applied with the Matlab® Sofware. Data were simulated according to the posteriori density of Bayesian statistics in order to obtain the confidence intervals of the different odds ratios.

## Results

### HLA-DRB1 Alleles Associated with RA

Seventy one percent of patients and 41% of controls were positive for the HLA-DRB1 shared epitope. Fifty one percent of patients and 37% of controls expressed one shared epitope positive allele. Twenty percent of patients and 4.5% of controls expressed two shared epitope positive HLA-DRB1 alleles.

RA associated HLA-DRB1 alleles were, by order of Odds Ratios, HLA-DRB1*04∶08 (OR = 10.3), HLA-DRB1*04∶01 (OR = 3.3), HLA-DRB1*04∶05 (OR = 3.3), HLA-DRB1*10 (OR = 2.9), HLA-DRB1*04∶04 (OR = 2.8), HLA-DRB1*09 (OR = 2.1), HLA-DRB1*01 (OR = 1.5) (Table 2). For genotype risk analysis, HLA-DRB1*04∶04, HLA-DRB1*04∶05, HLA-DRB1*04∶08 were pooled into the HLA-DRB1* 04SE group.

**Table 2:** HLA-DRB1 allelic frequencies in patients and controls.

	Patient numbern = 1714	Frequence (%)	Controlnumbern = 4356	Frequence (%)	Oddsratio	P-value(FISHER TEST)	Confidenceinterval
DRB1*01:XX	263	15.34	495	11.36	1.52*	6.6×10^−7^	1.28–1.79
DRB1*03:XX	101	5.89	470	10.79	0.51*	1.1×10^−9^	0.41–0.64
DRB1*04∶01	246	14.35	208	4.78	3.31*	5.1×10^−33^	2.71–4.04
DRB1*04∶02	18	1.05	38	0.87	1.2	0.55	0.64–2.17
DRB1*04∶03	18	1.05	63	1.45	0.7	0.31	0.41–1.27
DRB1*04∶04	120	7.00	114	2.62	2.80*	3.6×10^−14^	2.13–3.67
DRB1*04∶05	69	4.03	55	1.26	3.27*	1.12×10^−10^	2.26–4.78
DRB1*04∶06	1	0.06	8	0.18	0.31	0.46	0.007–2.37
DRB1*04∶07	10	0.58	41	0.94	0.55	0.11	0.23–1.16
DRB1*04∶08	32	1.87	8	0.18	10.33*	1.34×10^−11^	4.70–26.0
DRB1*07:XX	180	10.50	654	15.01	0.66*	2.31×10^−6^	0.55–0.79
DRB1*08:XX	21	1.23	123	2.82	0.42*	0.0001	0.25–0.68
DRB1*09:XX	30	1.75	37	0.85	2.07*	0.0038	1.24–3.47
DRB1*10:XX	56	3.27	50	1.15	2.9*	8.5×10^−8^	1.94–4.36
DRB1*11:XX	142	8.28	626	14.37	0.54*	3.3×10^−11^	0.44–0.65
DRB1*12:XX	21	1.23	71	1.63	0.7	0.29	0.43–1.23
DRB1*13:XX	130	7.58	542	12.44	0,58*	2.63×10^−8^	0.47–0.70
DRB1*14:XX^1^	39	2.28	169	3.88	0.58*	0.0016	0.39–0.82
DRB1*15:XX	161	9.39	465	10.67	0.87	0.14	0.7–1.05
DRB1*16:XX	56	3.27	119	2.73	1.20	0.26	0.85–1.67

* statistically significant.

ORs and p values by Fisher's test.

Alleles significantly underrepresented in RA patients (thus potentially protective) were HLA-DRB1*08 (OR = 0.42), HLA-DRB1*13 (OR = 0.58), HLA-DRB1*03 (OR = 0.51), HLA-DRB1*11 (OR = 0.54), HLA-DRB1*14 (OR = 0.58), HLA-DRB1*07 (OR = 0.66) (Table 2).

### HLA-DRB1 Genotypes and the Risk for Developing ACPA Positive RA

**Figure 1:**
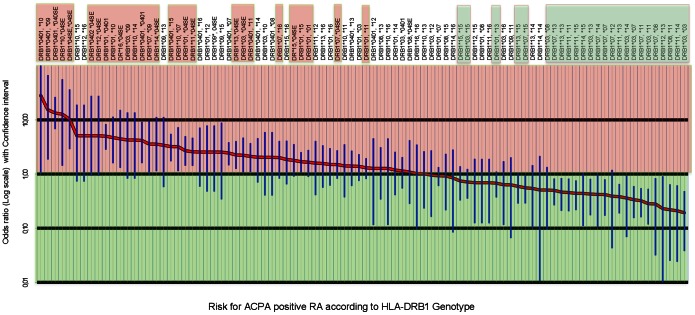
ORs could be calculated for 102 out of 136 possible HLA-DR genotypes. Here (for clarity) we only represent the ORs for the 91 most common (at least 10 patients plus controls). Genotypes highlighted in red have ORs significantly higher than 1, genotypes highlighted in green have ORs significantly lower than 1, genotypes non highlighted: OR not significantly different from 1.

**Figure 2:**
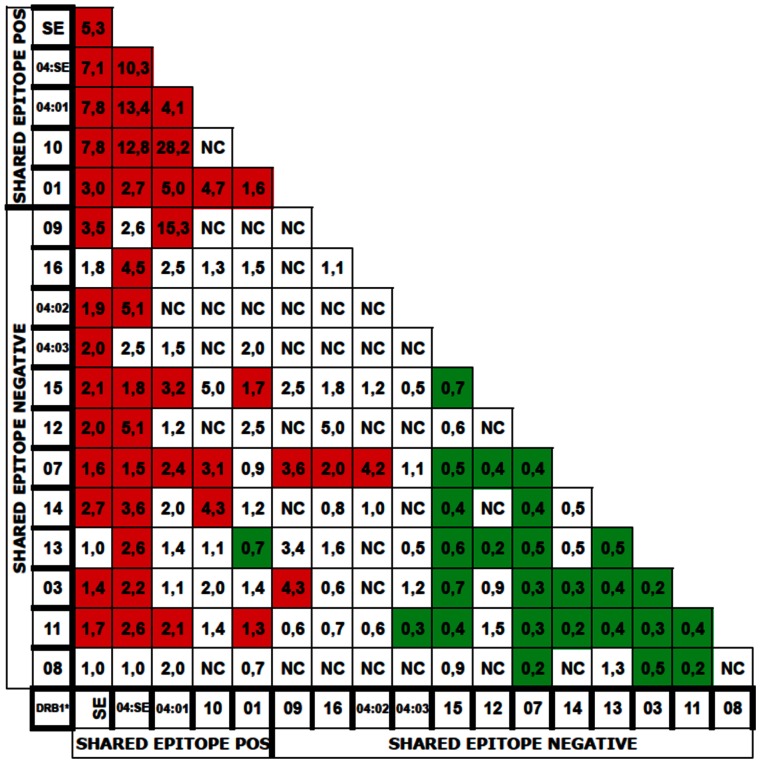
HLA-DRB1 genotype risk for ACPA positive RA. Red boxes: OR significantly higher than 1. Green boxes: OR significantly less than 1.

Sixteen HLA-DRB1 allelic groups define 136 genotypes. We arbitrarily decided not to calculate Odds Ratios for genotypes for which the total number of subjects (patients plus controls) was less than 5. This allowed us calculate Odds Ratios for 102 of 136 possible genotypes (Figure 1). Genotypic Odds Ratios ranged from 28 (HLA-DRB1*04∶01/*10) to 0.19 (HLA-DRB1*03/*03) (Figure 1). Thirty genotypes (29%) had ORs significantly higher than 1 and were considered “high risk”, 45 (44%) had ORs not significantly different from 1 and were considered neutral, 27 (26%) had ORs significantly lower than 1 and were considered “low risk” (Figure 2).

### High Risk and Low Risk Genotypes

Of 30 “high risk” genotypes, 18 contain one and 10 contain two RA associated HLA-DRB1 alleles. Only two: HLA-DRB1*07/*16 and HLA-DRB1*07/*04∶02 do not contain any RA associated allele (Figure 2).

Of 27 “low risk” genotypes, 26 contain no RA associated allele. Only HLA-DRB1* 01/*13 contains RA associated HLA-DRB1*01 (Figure 2).

**Figure 3:**
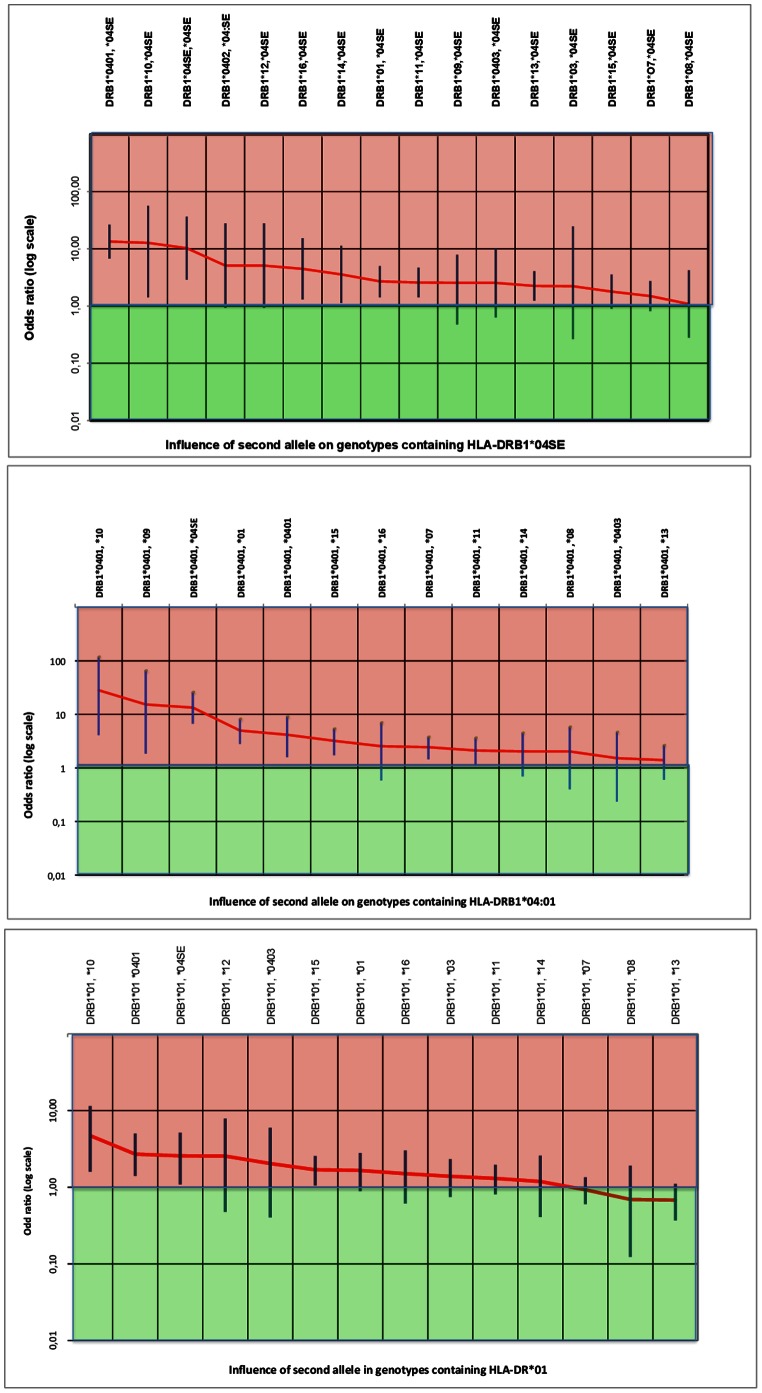
Influence of second allele on genotypic ORs in genotypes with HLA-DRB1*04SE, HLA-DRB1*04∶01, HLA-DRB1*01. Genotypes with HLA-DRB1*04SE and HLA-DRB1*04∶01 keep high ORs despite modulation by second allele.

Risk carried by RA associated allele is modulated by second allele in genotype as observed in genotypes containing HLA-DRB1*04SE, HLA-DRB1*04∶01, HLA-DRB1*01. For instance, ORs associated with HLA-DRB1*04SE genotypes range from 13.4 (HLA-DRB1*04SE/04∶01) to 1 (HLA-DRB1*04SE/08) (Figure 3).

## Discussion

### RA Associated and Non RA Associated HLA-DRB1 Alleles

In this study of HLA-DRB1 alleles expressed in 857 patients with ACPA positive RA and 2178 normal controls from South Eastern France, we confirm the classical association of RA with shared epitope positive HLA-DRB1 alleles.

Seven HLA-DRB1 alleles are associated with RA in our population, HLA-DRB1*04∶01, HLA-DRB1*04∶04, HLA-DRB1*04∶05, HLA-DRB1*04∶08, HLA-DRB1*01, HLA-DRB1*10, HLA-DRB1*09. HLA-DRB1*04∶01, HLA-DRB1*04∶04, HLA-DRB1*04∶08 are classically associated with RA in North and West European populations. HLA-DRB1*01∶01, DRB1*04∶05, DRB1*10 are more commonly associated with RA in South European, mediterranean populations [10]. Positive association of HLA-DRB1*09 with RA is less common. HLA-DRB1*09, with an RRRAE third hypervariable region motif, is associated with RA in Asians and in the UK [11]. It is not a stricto sensu shared epitope positive allele. Six alleles are negatively associated with RA in this study, HLA-DRB1*08, HLA-DRB1*11, HLA-DRB1*03, HLA-DRB1*13, HLA-DRB1*14 (DRB1*14∶02, a rare, shared epitope positive subtype of DRB1*14, was not found in our patients or controls), HLA-DRB1*07. Four of these, HLA-DRB1*07, HLA-DRB1*08, HLA-DRB1*11 and HLA-DRB1*13 are classified as protective or neutral in the Reviron model [4]. Surprisingly, HLA-DRB1*04∶02, present in 2% of patients and controls, and which was the first “protective“ allele to be identified [3], [4], is not negatively associated with RA (p = 0.54). Its frequency is almost identical in patients and controls.

### High Risk, Neutral and Low Risk HLA-DRB1 Genotypes

Genotypic risk depends on both HLA-DRB1 alleles in genotype. However, RA associated and non RA associated alleles do not have the same weight on genotypic risk. This study suggests that there are susceptibility alleles of unequal strength. The group of alleles called DRB1*04SE seems to be the strongest, almost always imposing susceptibility on genotype. Susceptible alleles like HLA-DRB1*04∶01 and HLA-DRB1*01 do not show such dominance (Figure 2). At the opposite end, genotypes with HLA-DRB1*07, HLA-DRB1*08, HLA-DRB1*11, HLA-DRB1*13, HLA-DRB1*03 are more often low risk than high risk or neutral. When associated with HLA-DRB1*04SE or HLA-DRB1*04∶01, these alleles lower the OR but not under 1. For instance, the ORs of genotypes with HLA-DRB1*04∶01 range from 13.4 (6,7–28) for the highly susceptible HLA-DRB1*04∶01/*04SE to 4.2 (1.59–9.22) for the homozygous genotype HLA-DRB1*04∶01/*04∶01 down to 2.44 (1.45–3.9) for HLA-DRB1*04∶01/*07, 2.1 (1.08–3.7) for HLA-DRB1*04∶01/11, 2 (0.4–5.9) for HLA-DRB1*04∶01/*08, 1.4 (0.6–2.7) for HLA-DRB1*04∶01/*13, 1.10 (0.5–2.1) for HLA-DRB1*04∶01/*03 (Figure 3). Similar Odds Ratio reduction by the same alleles is seen in genotypes with HLA-DRB1*04SE and HLA-DRB1*01. However, the significance of OR reduction by HLA-DRB1*07, HLA-DRB1*08, HLA-DRB1*11, HLA-DRB1*13 is limited by the width of confidence intervals for each genotype.

Figure 2 indicates the ORs associated with HLA-DRB1 genotypes. It is striking that 14 of the 15 SE double dose genotypes, in the upper left part of the table, have ORs higher than 1. Similarly, 25 of the SE negative genotypes, in the lower right part of the table, have ORs lower than 1. This pattern does not suggest the existence of protective alleles, but points to the influence of the number of copies of the shared epitope in determining the genotypic risk.

### Compatibility with the Reviron, du Montcel, and Shared Epitope Models

In 2001, we proposed a model (known as the Reviron model) to explain the influence of HLA-DRB1 alleles on the risk for developing RA [4]. This model was based on the classification of HLA-DRB1 alleles into 3 groups according to the charge of their P4 pocket. Shared epitope positive alleles have a very positively charged P4 pocket and are considered high risk. They include HLA-DRB1*04∶01, HLA-DRB1*04∶04, HLA-DRB1*04∶05, HLA-DRB1*04∶08 (here grouped as HLA-DRB1*04SE), HLA-DRB1*01, HLA-DRB1*10. Shared epitope negative alleles with a positively charged P4 pocket are considered neutral. They include HLA-DRB1*03, HLA-DRB1*09, HLA-DRB1*14, HLA-DRB1*15, HLA-DRB1*16, HLA-DRB1*04∶03 and most HLA-DRB1*11. Shared epitope negative alleles with a negative or neutral P4 pocket are considered protective. They include HLA-DRB1*04∶02, HLA-DRB1*07, HLA-DRB1*08, and most HLA-DRB1*13. Some of the experimental data here diverge from the Reviron classification. We find that HLA-DRB1*09, predicted as neutral, is associated with RA. We also find that the “protective” allele HLA-DRB1*04∶02, is not negatively associated with RA.

Another model known as the du Montcel model, just distinguishes 3 categories of alleles: very high risk (S2/HLA-DRB1*04∶01), high risk (S3P/HLA-DRB1*04SE, HLA-DRB1*01, HLA-DRB1*10) and neutral (S1 (including HLA-DRB1*0402) S3D and X/all others, including HLA-DRB1*09) [5]. Regarding the neutral status of HLA-DRB1*04∶02, the du Montcel classification matches our observations. Regarding the status of HLA-DRB1*09, an RA associated allele in our study, the du Montcel classification does not match our results. Finally, the du Montcel classification identifies HLA-DRB1*0401 as the highest risk allele, whereas in our study, HLA-DRB1*04SE seems to contribute higher risk.

Our data confirm the shared epitope hypothesis and its already known “dose effect”. Indeed, if one considers classical shared epitope positive alleles HLA-DRB1*04SE, *04∶01, *10 and *01, they define 10 “double dose” genotypes. Only 9 of these 10 genotypes have been considered for OR calculation (the homozygous DRB1*10 subjects were less than 5). All 9 had ORs significantly higher than 1 (high risk genotypes) (Figure 2). Among 48 “single dose” shared epitope positive genotypes, 40 were considered for OR calculations, of which 17 (40%) were “high risk”, 22 were neutral and only 1, DRB1 *13/DRB1 *01, was “low risk”. Finally, among 78 shared epitope negative genotypes, 53 were considered for OR calculation, of which 25 (47%) were “low risk”, 24 (45%) were “neutral” and 4 (8%)” high risk” (Figure 2). In short, we find that every “double dose“, and 40% of “single dose” shared epitope positive genotypes are high risk, against only 8% of shared epitope negative genotypes. If one considers ORs associated with pooled shared epitope positive genotypes (genotypes obtained by considering shared epitope positive alleles as one category), then 14 of 17 pooled shared epitope positive genotypes are high risk and 3 are neutral (Figure 2). Altogether, these data confirm validity of the shared epitope as the determinant of HLA-DRB1 susceptibility for developing RA. However, classifications of alleles according to models are based on a priori assumptions of how alleles interact and determine risk (control of peptide binding…). Here, we made no such assumption and just calculated Odds ratios for as many genotypes as we could. We provide a table that gives an idea of the risk for developing ACPA positive RA given one’s HLA-DR genotype. Under its current form, it gives the risk for 102 of 136 genotypes, accounting for 97% of our population (Figure 2). It may be used to help diagnosis of undifferentiated arthritis, especially before the emergence of ACPA response. It may also prove useful for genetic counseling in families affected by rheumatoid arthritis, indicating which individual to monitor for emergence of ACPA response and possibly early treatment or even prevention.

Finally, our genotypic risk table does not necessarily reflects the effect and interaction of HLA-DRB1 genes only, but may also involve non HLA-DRB1 genes on HLA-DRB1 haplotypes.

### The Remaining Third: ACPA Negative RA

This study did not approach the very controversial issue of ACPA negative RA and its HLA-DRB1 association(s). Indeed, ACPA negative RA is somewhat heterogeneous and careful analysis of patients’ files often ends up with diagnosis other than RA. Still, one large scale study of HLA-DRB1 associations in ACPA negative RA patients suggests that half of them test positive for rheumatoid factors and show HLA-DRB1 association similar to ACPA positive RA [11]. Such patients will be the objects of future studies.

### Other Polymorphisms in the HLA Region may be Relevant to Susceptibility for Developing ACPA Positive RA

A recent very large-scale association study using 3000 SNPs in the MHC confirmed the importance of polymorphisms in the HLA-DRB1 chain, pinpointing three polymorphic residues, two of which are included in the shared epitope region. However, this study did not consider the genotypic effect, that is how BOTH HLA-DRB1 alleles interact to modulate risk for developing RA [12]. Here, we did not focus on the identification of new polymorphic residues in HLA-DRB1 alleles but on the interaction of individual alleles inside genotypes to influence RA susceptibility.
